# A Review of Countermovement and Squat Jump Testing Methods in the Context of Public Health Examination in Adolescence: Reliability and Feasibility of Current Testing Procedures

**DOI:** 10.3389/fphys.2019.01384

**Published:** 2019-11-07

**Authors:** Luca Petrigna, Bettina Karsten, Giuseppe Marcolin, Antonio Paoli, Giuseppe D’Antona, Antonio Palma, Antonino Bianco

**Affiliations:** ^1^Ph.D. Program in Health Promotion and Cognitive Sciences, Department of Psychology, Educational Science and Human Movement, University of Palermo, Palermo, Italy; ^2^Department of Exercise and Sport Science, LUNEX International University of Health, Exercise and Sports, Differdange, Luxembourg; ^3^Department of Biomedical Sciences, University of Padova, Padua, Italy; ^4^CRIAMS-Sport Medicine Centre, University of Pavia, Pavia, Italy; ^5^Department of Psychology, Educational Science and Human Movement, University of Palermo, Palermo, Italy; ^6^Regional Sport School of CONI Sicilia, Italian National Olympic Committee, Sicilia, Italy

**Keywords:** vertical jump, CMJ, SJ, standardized protocol, adolescent, public health, standard operating procedure, physical fitness

## Abstract

**Background:**

In the context of a public health physical fitness (PF) examination in adolescence, a countermovement jump (CMJ) and a squat jump (SJ) are two vertical jump (VJ) tests widely used to evaluate lower limb muscle strength and power, respectively. The main criticism of both the CMJ and SJ test is the lack of test standardization. Therefore, the objectives of this review are: (a) to gather information about both jumps; (b) to investigate whether it is possible to identify common procedures referred to in the CMJ and SJ technical execution, and (c) to design standard operating procedures (SOPs) to promote CMJ and SJ standardization in an adolescent population aged 12–18 years.

**Methods:**

The review partially adopted the Preferred Reporting Items for Systematic Reviews and Meta-Analyses Statement (PRISMA). Due to growing attention in monitoring physical health through field tests in recent years, articles were collected using the PubMed, Web of Science, and Scopus databases from January 2009 to July 2019. Original articles in which CMJ or SJ were used to assess the muscular strength in adolescents were eligible for further analysis. Articles written in English was imposed as a limit.

**Results:**

A total of 117 studies met the inclusion criteria. The description of the CMJ and SJ test procedures was different within the literature, with discrepancies in the jump technique, number of jumps, and measurement devices used.

**Conclusions:**

A lack of method standardization for both the CMJ and the SJ test was identified. Based on the literature, SOPs for both VJs were proposed. These are useful in the context of public health PF examination in adolescents, as they facilitate an unbiased comparison of jump performance data between published studies.

## Introduction

Muscular strength and power, cardiorespiratory endurance, body composition, and flexibility are health-related attributes of physical fitness (PF) (Caspersen et al., 1985) and consequently are considered key health status markers in humans (Catley and Tomkinson, 2013). To prevent pathologies and diseases that occur during adulthood, monitoring PF during adolescence is, therefore, important (Ortega et al., 2008b). In this context, the identification of children who are not developing healthy fitness habits using valid, reliable but also feasible measurement tools is essential (Davis et al., 2008; Faigenbaum et al., 2011; Garber et al., 2011). In the last decade, several research groups have focused their work on assessing the health status of children and adolescents, developing test batteries such as the ALPHA health-related fitness test battery (Ruiz et al., 2011), the ASSO project (Bianco et al., 2015), and the PREFIT battery (Ortega et al., 2015).

Physical fitness can be objectively and accurately measured through laboratory and field tests (Cooper, 1968; Astrand, 1976; Boone et al., 1978; Leger and Lambert, 1982; Inbar et al., 1996; Mayorga-Vega et al., 2014). Laboratory tests are generally more reliable, while field tests are commonly less expensive and more comfortable to administer (Heyward, 1991), and importantly they are characterized by a high level of ecological validity. Therefore, field tests are more suitable in population-based studies, especially in a school or college setting (Artero et al., 2011). Some field tests are subject to a standardized testing procedure, for example, the Cooper test (Cooper, 1968); the 20-m Shuttle Run Test (Leger and Lambert, 1982), or the sit-and-reach test (Wells and Evelyn, 1952). Conversely, to date the vertical jump (VJ) test does not consist of such standardized testing procedures.

Vertical jumping is a multi-joint movement that requires complex motor coordination, and it has been identified as one of the fundamental movement skills (Gallahue, 2002). VJ tests are widely used to evaluate simple and complex tasks (Suchomel et al., 2016), such as sprint acceleration, sprint deceleration, throwing (Manno, 2008; Comfort et al., 2012a, b; Seitz et al., 2014), and change of direction (Nimphius et al., 2010; Spiteri et al., 2014; Suchomel et al., 2016). Furthermore, to evaluate lower limb muscular strength and due to the simplicity and richness in outcome information, VJ tests are widely used by strength and conditioning professionals, coaches, and health care professionals (Liebermann and Katz, 2003; Duthie, 2006).

The countermovement jump and squat jump (CMJ and SJ, respectively) are two examples of VJs which are both derived from the Sargent jump (Sargent, 1921, 1924). Both CMJ and SJ are considered reliable and valid (Markovic et al., 2004) in children (Fernandez-Santos et al., 2015). The CMJ is characterized by an initial countermovement (CM) before the toe-off phase (Bobbert et al., 1996), and the CMJ provides information about the reactive strength of the lower limbs (Young, 1995). In contrast, the SJ starts from a stationary, semi-squatting position and it provides information about leg power performance (Anderson and Pandy, 1993; Young, 1995). In sedentary individuals, as well as in elite athletes, the resultant jump height is correlated with explosive muscle strength (Sargent, 1921, 1924; Bosco and Komi, 1979, 1980; Bosco and Viitasalo, 1982) and with performance components, such as speed (Wisloff et al., 2004), agility (Barnes et al., 2007), and power (Liebermann and Katz, 2003; Markovic et al., 2004; Patterson and Peterson, 2004; Tricoli et al., 2005). The SJ performance is also considered a measure of coordinated activities (Tomioka et al., 2001; Eloranta, 2003; Myer et al., 2005). In this context, Van Hooren and Zolotarjova (2017) in a recent review highlighted the differences between CMJ and SJ performances, emphasizing the need for future research to investigate the exact interaction of the mechanisms that explain the difference between the two jumps.

The lack of robust and consistent testing methods for CMJ and SJ evident in the literature compromises the quality of the research in this area (Eagles et al., 2015). Eagles et al. (2015) in their meta-analysis on VJ tests stated a lack of standardization in jump phase identification (i.e., starting, push-off, toe-off, and apex of the jump phase) which results in notable differences in the duration of the jump phases, the time to reach peak force, and in the rate of force development. Fitzgerald et al. (2018) suggested the use of the SJ to bypass the problem of the identification of the related phases, as this jump comprises of less variables than other kinds of jumps. Van Praagh and Dore (2002) stated that there is a distinct need to create a standardized jump protocol. Furthermore, some researchers advocate the use of standard operating procedures (SOPs) as being superior to the teaching of “laboratory manuals,” in that SOPs provide a step-by-step guide to the details related to a process which allows for the exact replication of all steps involved when repeating the process (Angiuoli et al., 2008; Tuck et al., 2009). It is important to note that SOPs are widely adopted in many other areas (Angiuoli et al., 2008), such as biology (Roseti et al., 2015) or medicine, for example in stroke prevention and treatment (Ntaios et al., 2015), critical illness (Sherren et al., 2014), or pre-hospital critical care interventions (Rognas et al., 2013).

To the best of our knowledge, no research to-date has stipulated clear guidelines for the CMJ and SJ tests. In fact, published works used different testing procedures, without specifying some essential parts for the replicability of the work.

### Objectives

The first objective of this review was to gather information about testing methods used in research related to the assessment of PF, specifically muscular strength, using CMJ and SJ performance tests in adolescence. The second objective was to investigate if there are standard aspects between these CMJ and SJ testing methods and to identify the most common ones used. If these were not evident, the third objective was to develop SOPs considering: the jump phases; the devices used, and the number of jumps performed. As described by Bobbert et al. (1996), for a better understanding of the differences between the CMJ and the SJ, it is useful to divide both VJs into phases (e.g., starting position, the start of the push-off, the toe-off, and the apex of the jump phase).

### Research Question

With a particular view of the adolescent population, the research question addressed whether there are clearly defined SOPs for the CMJ and the SJ test evident in the literature and then, in parallel, to gather information about both VJs.

## Materials and Methods

### Study Design

This review of literature partially adopted the Preferred Reporting Items for Systematic Reviews and Meta-Analyses (PRISMA) statement (Moher et al., 2009). The following key points were not used: protocol and registration (5), data items (11), risk of bias in individual studies (12), summary measures (13), synthesis of results (14), risk of bias across studies (15, 22), additional analyses (16, 23), risk of bias within studies (19), results of individual studies (20), and synthesis of results (21).

### Participants, Interventions, Comparators

Population, Intervention, Comparison, Outcomes, Study design criteria (PICO-S criteria) described in PRISMA (Moher et al., 2009) were considered, to include and exclude research publications. The population under examination were adolescents. According to Radnor et al. (2018), adolescent females include an age range between 12 and 18 years, and for males this age range is between 14 and 18 years. A population between 12 and 18 years old, of both genders with no particular conditions (i.e., mental disease or physical problems), was considered in the present work, to avoid misunderstandings between the different gender age ranges. Children, adults, the elderly, and elite athletes (due to the possible adaptation of the VJs for the sport characteristic) were excluded. As the authors wanted to examine the jump testing method used, interventions, comparisons, and outcomes of the studies were not considered as inclusion or exclusion criteria. Regarding the study design, original articles were eligible for further analysis in which CMJ or SJ performance tests were used to assess lower limb muscular strength as part of PF evaluations in adolescents and not as training interventions. Due to the risk of involving other populations that were not adolescents, longitudinal studies were excluded.

No restriction criteria were applied for the country of origin, but only works written in English were considered. Reviews, meta-analyses, abstracts, citations from scientific conferences, statements, opinion pieces, commentaries, editorials, letters, book reviews, books, and non-peer reviewed journal articles were excluded.

### Search Strategy

The databases consulted for relevant original articles were PubMed (NLM), Web of Science (TS), and Scopus. The search strategy included the use of the terms in the search field “title” and “topic” of each database. The terms used were divided into three groups. Group A used the following keywords: “countermovement jump^∗^,” “squat jump^∗^,” and “vertical jump^∗^.” Group B used the following keywords: “maximal dynamic strength,” “field-based physical fitness test^∗^,” “fitness-test battery^∗^,” “field test^∗^,” “physical fitness,” “muscle strength,” “strength,” “resistance training,” “physical education,” “reliability,” and “validity.” Finally, group C used the keywords: “youth,” “preadolescence^∗^,” “adolescent^∗^,” “public health,” and “health promotion.” For each database, term by term of Group A was matched with each term of Group B and Group C using a Boolean operator (AND).

### Data Sources, Studies Sections, and Data Extraction

**FIGURE 1:**
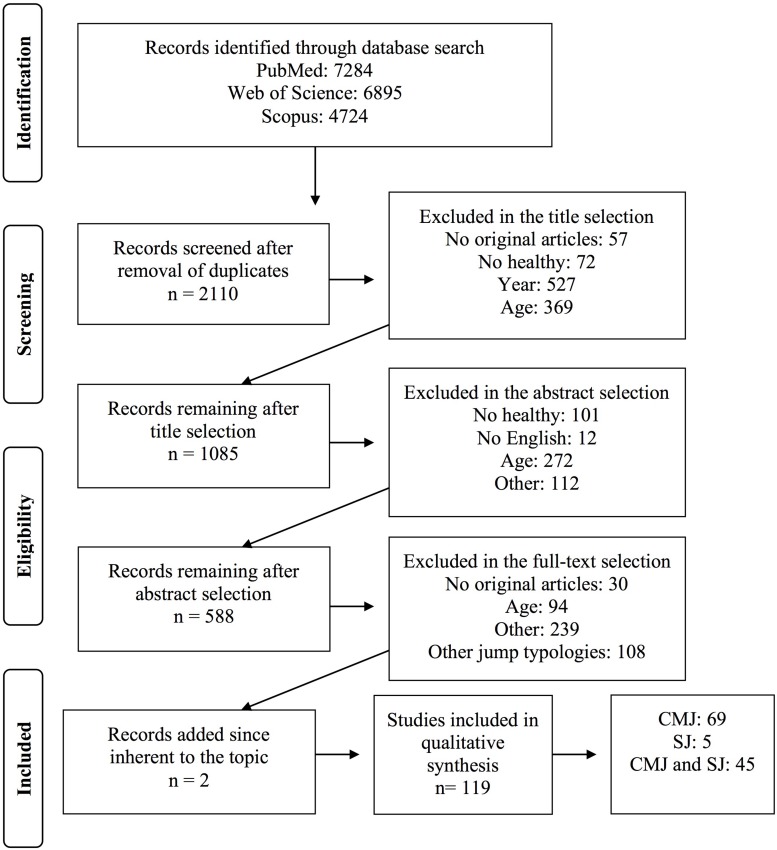
Flow diagram representing the steps applied in the selection process of manuscripts.

Due to the growing interest in monitoring PF in young people in the last 10 years (Ruiz et al., 2011; Bianco et al., 2015; Ortega et al., 2015), databases were searched for studies published between the 1 January 2009 up to the 8 July 2019. All original articles selected from databases were transferred to the EndNote X8 software to check the presence of duplicates. In a second screening phase, two investigators, working independently, selected the articles against the inclusion and exclusion criteria described in the section “Participants, Interventions, Comparators,” with a three steps process: (i) selection based on the titles; (ii) selection based on the abstract; and (iii) selection based on the full text. If there was disagreement between the two investigators, a third investigator took the final decision. A flow diagram that summarizes the selection process is reported in Figure 1.

The following information was extracted: first author, year of publication, sample size, participants’ age (range, mean, and standard deviation), gender, aim of the study, active or sedentary, physical or sport activities practiced, jump method used, device employed, and main results. The information extracted from any section of the manuscript was consequently recorded in table format and descriptively summarized.

Following this stage, a descriptive analysis of the CMJ and SJ test technique was performed, and all common aspects between the VJs were considered.

## Results

### Study Selection and Characteristics

After duplicate removal, title, abstract, and full-text comparison against inclusion and exclusion criteria, the number of publications was reduced from 18,903 to 1,860 with a final total of 119 articles being included. More details are provided in the diagram flow presented in Figure 1.

The 119 original studies were divided into subgroups as follows:

Sixty-nine studies solely using the CMJ test to evaluate muscular strength in adolescents (Table 1);

**TABLE 1:** General information containing the CMJ test only.

**Study**	**Sample**	**Age**	**Age mean**	**Physical activity**	**Protocol**	**Device**
	**(female) [male]**	**range**	**(*SD*)**	**level**		
Alvarez-San Emeterio and Gonzalez-Badillo, 2010	(15) [16]	13–16	14.6 (1.1)	Ski	OP	CM
Alvarez-San Emeterio et al., 2011	(19) [20]	13–16	14.7 (1.2)	Ski/none	OP	CM
Amaro et al., 2017	[21]	–	12.7 (0.8)	Swim	Garrido et al., 2010	CM
Boccolini et al., 2013	[23]	14–15	14.8 (0.1)	Basketball	Bosco et al., 1983	PS
Brännström et al., 2017	(19)	13–16	15.3 (0.7)	Soccer	OP	PS
Brunelli et al., 2014	[11]	–	13.3 (0.6)	Basketball	Breed and Young, 2003	CM
Bubanj et al., 2018	[60]	17–18		Mixed	No info	ABS
Buchan et al., 2010	(25) [64]	–	16.7 (0.6)	School	No info	PS
Buchheit et al., 2010	18	–	15.8 (0.9)	School	OP	PS
Castagna et al., 2013	20	–	15.5 (0.8)	Rugby	Domire and Challis, 2007	ABS, PS, FP
Chaouachi et al., 2017	[26]	13–14	13.9 (0.3)	Soccer	Chaouachi et al., 2014	CM
Claudino et al., 2016	[18]	14–17	15.2 (0.9)	Futsal	Ugrinowitsch et al., 2007	CM
Cortis et al., 2011	[10]	15–16	15.7 (0.2)	Basketball	OP	PS
Duncan et al., 2013	(51) [40]	12–16	14.3 (1.3)	None	OP	FP
Faigenbaum et al., 2010	[19]	–	16.5 (1.1)	Athlete	OP	Vanes
Franco-Márquez et al., 2015	[44]	14–18	14.7 (0.5)	Soccer	OP	PS
Gallo-Salazar et al., 2017	12	–	14.4 (0.9)	Tennis	Bosco et al., 1983	CF
Garcia-Pinillos et al., 2015	[43]	14–18	15.6 (1.5)	Soccer	OP	PS
Gavanda et al., 2019	[47]	–	17 (0.8)	Football	OP	PS
Gonzalez-Garcia et al., 2019	(24)	14–16	16.8 (1.6)	Soccer	OP	VA
Gonzalo-Skok et al., 2017	[30]	14–16	14.6 (0.5)	Basketball	OP	PS
Granacher et al., 2011	(15) [13]	–	16.7 (0.6)	School	OP	FP
Granacher and Gollhofer, 2011	28	–	16.8 (0.6)	None	OP	FP
Gorski et al., 2018	[31]	–	16.0 (0.2)	Handball	OP	FP
Hale et al., 2019	(15)	–	15.1 (2.7)	Volleyball	OP	Vanes
Hall et al., 2016	(20)	–	12.5 (1.7)	Gymnast	OP	JM
Harries et al., 2018	[16]	15–18	16.4 (1)	Rugby	Cormack et al., 2008	LPT
Hydren et al., 2013	(7) [4]	–	13.7 (0.5)	Ski	OP	Timing pad
Holden et al., 2015	(84) [97]	–	13 (1.4)	Mixed	OP	VA
Kinugasa and Kilding, 2009	28	–	14.3 (0.7)	Soccer	OP	Vanes
Klusemann et al., 2012	(21) [17]	–	14 (1)	Basketball	OP	FP
Idrizovic et al., 2018	(47)	–	16.6 (0.6)	Volleyball	OP	FP
Imai et al., 2014	[27]	–	16.3 (0.5)	Soccer	OP	Mat switch
Lago-Penas et al., 2014	[156]	13–15	15 (2)	Soccer	OP	CM
Lehnert et al., 2013	16	–	16.7 (0.7)	Soccer	OP	FP
Lyle et al., 2015	(14) [15]	–	16.1 (0.8)	Soccer	OP	Vanes
Marques et al., 2013	[52]	–	13.4 (1.4)	Soccer	Wisloff et al., 2004	CM
Marques et al., 2016	[167]	13–18	15.7 (1.7)	Soccer	Marques et al., 2008	TC
Martin et al., 2019	(37) [77]	–	16.6 (1.1)	Mixed	OP	Vanes
Matthys et al., 2013	472	13–16	13.5 (0.3)	Handball	OP	PS
McCormick et al., 2016	(14)	–	16 (0.8)	Basketball	OP	Vanes
Moraes et al., 2013	[38]	14–18	15.5 (1)	None	Castro-Pinero et al., 2009	Vanes
Muehlbauer et al., 2012	(15) [13]	16–17	16.7 (0.7)	None	OP	FP
Munivrana et al., 2015	(152) [154]	15–18	16.9 (1.7)	Tennis	OP	PS
Negra et al., 2016	24	–	12.8 (0.2)	Soccer	OP	PS
Negra et al., 2017	[37]	–	12.1 (0.5)	Soccer	OP	PS
Oliver et al., 2015	[11]	–	16.9 (0.8)	Rugby	Lloyd et al., 2009, 2011	CM
Padulo et al., 2015b	[17]	–	16	Soccer	Bosco et al., 1982	PS
Paradisis et al., 2014	47	–	14.6 (1.7)	Active	OP	Vanes
Paul et al., 2019	[19]	–	16.2 (0.8)	Soccer	OP	PS
Perroni et al., 2017	[112]	12–19	14 (2)	Soccer	Bosco et al., 1983	PS
Quagliarella et al., 2011	[123]	13–18	15.7 (1.4)	Soccer	OP	FP
Saez de Villarreal et al., 2015	26	14–15	15.1 (0.2)	Soccer	OP	CF
Sanchez-Urena et al., 2017	[10]	14–15	14 (0.4)	Basketball	Bosco et al., 1983	PS
Sawczuk et al., 2017	(20) [39]	–	17.3 (0.7)	Sportive	OP	PS
Sekulic et al., 2014	[84]	12–13	15.2 (1.3)	None	OP	PS
Smart and Gill, 2013	82	13–18	15.2 (1.3)	Rugby	OP	Vanes
Struzik et al., 2017	(151) [154]	12–16	14.4 (0.8)	Mixed	OP	FP
Takai et al., 2013	[94]	12–16	13.7 (0.6)	None	Bosco et al., 1983	FP
Thomas et al., 2017b	[16]	–	17.3 (0.6)	Basketball	OP	FP
Till and Jones, 2015	[121]	12–16	14.4 (1.7)	Rugby	OP	JM
Tishukaj et al., 2017	(159) [195]	–	14.5 (0.4)	None	Castro-Pinero et al., 2009	FP
Torres-Luque et al., 2015	146	14–17	14.6 (1.1)	Judo	Aragon-Vargas, 2000	CF
Turner et al., 2017	(33) [46]	–	15.9 (0.7)	Fencer	OP	PS
Uthoff et al., 2018	[43]	13–15	14.6 (0.3)	Mixed	OP	Vanes
Weakley et al., 2017	[35]	–	16.9 (0.4)	Rugby	OP	FP
Wong et al., 2009	[70]	–	13.4 (0.7)	Soccer	OP	JM
Wong et al., 2010	[62]	13–14	13.7 (0.5)	Soccer	OP	JM
Yanci et al., 2016	(36) [28]	13–15	14.08 (1)	Mixed	Maulder and Cronin, 2005	PS

*ABS, accelerometer-based system; CF, contact platform; CM, contact mat; FP, force plate; JM, jump mat; LPT, linear-position transducer; OP, own protocol; PA, physical activity; PF, physical fitness; PP, physical performance; PS, photoelectric system; VA, video analysis; VJ, vertical jump.*

Five studies solely using the SJ test to evaluate muscular strength in adolescents (Table 2);

**TABLE 2:** General information containing the SJ test only.

**Study**	**Sample (female) [male]**	**Age range**	**Age mean (*SD*)**	**Physical activity level**	**Protocol**	**Device**
Dayne et al., 2011	[11]	–	15.6 (0.5)	Mixed	OP	FP
Fischetti et al., 2019	[24]	12–14	13.2 (0.8)	Mixed	OP	ABS
Fischetti et al., 2018	[22]	13–14	13.6 (0.5)	Mixed	OP	ABS
Maciejewski et al., 2018	[14]	–	15.3 (0.6)	Rowers	OP	VA
Radnor et al., 2017	[8]	12–16	12.6 (0.2)	None	Lloyd et al., 2009	CM

*ABS, accelerometer-based system; CM, contact mat; FP, force plate; OP, own protocol; VA, video analysis.*

Forty-five studies where both the CMJ and the SJ test were employed to evaluate muscular strength in adolescents (Table 3).

**TABLE 3:** General information containing both, CMJ and the SJ tests.

**Study**	**Sample**	**Age**	**Age mean**	**Physical activity**	**Protocol**	**Device**
	**(female) [male]**	**range**	**(*SD*)**	**level**		
Alberti et al., 2014	[81]	–	16.9 (5.4)	Basketball	Bosco et al., 1983	PS
Aloui et al., 2013	[12]	–	13.3 (0.4)	Soccer	OP	CM
Battaglia et al., 2014	(51)	14–15	15 (0.9)	Mixed	Moir et al., 2004	PS
BenOunis et al., 2013	[42]	–	14.8 (0.4)	Soccer	OP	PS
Borges et al., 2018	(25) [64]	12–17	14.5 (0.5)	Soccer	Bosco et al., 1983	CF
Bouteraa et al., 2018	(26)	–	16.4 (0.5)	Basketball	OP	PS
Çakir-Atabek, 2014	(11) [13]	–	15.8 (0.8)	Mixed	OP	FP
Carvalho et al., 2012	[16]	–	14.5 (2.8)	Tennis	OP	JM
Chelly et al., 2009	[22]	–	17 (0.4)	Soccer	OP	CM
Coelho et al., 2010	80	12–14	12.9 (0.3)	Mixed	Bosco et al., 1983	CM
Comfort et al., 2014	[34]	–	17.2 (0.6)	Soccer	OP	JM
Cunha et al., 2017	[46]	12–18	14.2	Soccer	OP	Jump plate
Daneshfar et al., 2018	20	–	16.4 (0.7)	Handball	Bosco and Rusko, 1983	ABS
Dowse et al., 2017	(12)	–	14.2 (1.9)	Dancer	OP	FP
Garcia-Pinillos et al., 2014	[30]	–	15.9 (1.4)	Soccer	OP	Jump sensor
Girard and Millet, 2009	[12]	–	13.6 (1.4)	Soccer	OP	PS
Greco et al., 2019	(56)	13–18	16.8 (0.6)	Volleyball	OP	VA
Grgantov et al., 2013	(56)	13–15	14.6 (0.5)	Volleyball	OP	PS
Hammami M. A. et al., 2013	[50]	–	14.4 (0.3)	Soccer/none	OP	PS
Hammami M. A. et al., 2017	[40]	–	14.4 (0.3)	Soccer	OP	PS
Hammami R. et al., 2018	[56]	12–14	13.9 (1.4)	Handball	OP	FP
Hammami M. et al., 2017	[44]	12–14	16 (0.5)	Soccer	OP	FP
Hammami M. et al., 2018	[31]	15–17	16 (0.5)	Soccer	OP	FP
Hespanhol et al., 2013	[110]	13–18	15 (0.8)	Mixed	Bosco, 1994	CM
Hoshikawa et al., 2013	[28]	13–14	12.7	Soccer	OP	VA
Lesinski et al., 2016	(19)	–	14.7 (0.6)	Soccer	OP	ABS, PS, FP
Loturco et al., 2016	[10]	–	17 (0.7)	Swim	OP	CF
Maio Alves et al., 2010	[23]	–	17.4 (0.6)	Soccer	OP	CM
Makhlouf et al., 2016	[57]	–	13.7 (0.5)	Soccer	OP	FP
Maly et al., 2015	[22]	–	13	Active	CMJ: OP; SJ: no info	FP
Moliner-Urdiales et al., 2010	(180) [183]	12–17	14.8 (1.2)	None	OP	CF
Nikolaidis and Knechtle, 2016	[12]	–	12.2 (0.5)	Soccer	Fernandez-Santos et al., 2015	PS
Ortega et al., 2011	(1845) [1583]	12–18	14.9 (1.2)	None	Ruiz et al., 2006; Ortega et al., 2008a	
Padulo et al., 2015c	[18]	–	16	Soccer	Bosco et al., 1983	PS
Padulo et al., 2015a	[18]	–	16.4 (0.5)	Basketball	Bosco and Rusko, 1983	ABS
Padulo et al., 2016a	[18]	–	16	Soccer	Bosco and Rusko, 1983; Bosco et al., 1983	PS
Padulo et al., 2016b	(22) [14]	–	16 (1)	Basketball	Bosco and Rusko, 1983	ABS
Pino-Ortega et al., 2018	[15]	–	14.7 (0.2)	Soccer	OP	CF, ABS
Pojskic et al., 2018	[20]	–	17 (0.9)	Soccer	OP	CM
Ramirez-Campillo et al., 2018	[18]	17–18	17.4 (0.8)	Soccer	Maulder and Cronin, 2005	CM
Santos and Janeira, 2011	[24]	16–17	14.7 (0.4)	Basketball	Bosco, 1994	CM
Santos and Janeira, 2012	[25]	14–15	14.5 (0.6)	Basketball	Bosco, 1994	CM
Secomb et al., 2015	(7) [23]	–	14.8 (1.7)	Surf	Hasson et al., 2004; McGuigan et al., 2006; Sheppard et al., 2008	FP
Thomas et al., 2017a	(26)	–	16.1 (1.2)	Netball	OP	JM
Yousfi et al., 2018	[14]	–	16.9 (0.7)	Combact	Chamari et al., 2004	PS

*ABS, accelerometer-based system; CF, contact platform; CM, contact mat; FP, force plate; JM, jump mat; OP, own protocol; PA, physical activity; PF, physical fitness; PP, physical performance; PS, photoelectric system; VA, video analysis; VJ, vertical jump.*

A total of 9,940 individuals were considered. Of this 34% (3,373) were females, 57% (5,630) males, and for the remaining 9% (937) gender was not specified. The mean age was 15.2 years.

### Synthesized Findings About Both Jumps

#### Study Characteristics for CMJ

There was no consistency in the description of the CMJ test method in the literature (Tables 1, 3). The protocol developed by Bosco et al. (1983) was the one most commonly adopted (Coelho et al., 2010; Boccolini et al., 2013; Takai et al., 2013; Alberti et al., 2014; Padulo et al., 2016a; Gallo-Salazar et al., 2017; Perroni et al., 2017; Sanchez-Urena et al., 2017; Borges et al., 2018). According to the instructions of this protocol, participants have to stay in an upright position before the execution of the VJ, which starts with a CM until the legs are bent down to 90°. A more precise description, with information of the knee angle during the standing position, the landing (180°), and the CM (reach approximately 90°) phases, is given by Fernandez-Santos et al. (2015), cited one time (Nikolaidis and Knechtle, 2016). Yanci et al. (2016) and Ramirez-Campillo et al. (2018) cited the protocol of Maulder and Cronin (2005), giving general information on the take-off and the landing phases, which both had to be executed with extended knees and ankle joints. Regarding the CM phase, the protocols of Cormack et al. (2008) allowed participants to self-select the CM depth. Information regarding the speed of the CM phases are given by Ortega et al. (2008a) and Chaouachi et al. (2014). In the protocol proposed by Maulder and Cronin (2005) participants were asked to “sink as quickly as possible” reaching a knee angle of approximately 120°, which was similar to Ortega et al. (2008a) who instructed participants to perform a fast CM. The protocol of McGuigan et al. (2006) cited by Secomb et al. (2015) standardized the position of the hands by requiring the participants to perform the jump while holding a light weight (1.0 kg) over the shoulders. The protocol of Castro-Pinero et al. (2009) was employed twice (Moraes et al., 2013; Tishukaj et al., 2017). Because of the use of the arms, it is different from the protocols previously described, as participants had to touch and mark a wall with their fingertips at a highest possible point. Finally, the protocol by Aragon-Vargas (2000) was the only report requesting that the CMJ be executed barefoot.

#### Study Characteristics for SJ

Likewise, no standardized jump method was detected for the SJ (Tables 2, 3). The protocol of Bosco et al. (1983) was used five times and required participants to perform the SJ from a half squat position with knees bent at 90°, torso straight, and both hands on their waist (Coelho et al., 2010; Alberti et al., 2014; Padulo et al., 2015c, 2016a; Borges et al., 2018). Additionally, three studies (Santos and Janeira, 2011, 2012; Hespanhol et al., 2013) used the 1983 protocol of Bosco (1994), but cited his work of 1994. The protocol of Lloyd et al. (2009) required the participants to take-off and land on the same spot. Furthermore, before the SJ test, some protocols instructed the participants to wait 4 (Maulder and Cronin, 2005), 3 (McGuigan et al., 2006), or 2 s (Lloyd et al., 2009) before executing the jump in order to control the assumed position. More information regarding the landing can be retrieved in the protocol of Fernandez-Santos et al. (2015). According to these researchers, the knees had to be kept extended at an angle of 180° and the ground contact during the landing had to be initiated with the toes. Straight legs in both take-off and landing was also used in the protocol of Maulder and Cronin (2005). Furthermore, according to Lloyd et al. (2009), the landing phase had to be performed with both legs fully extended while looking forward and, to maintain balance, to gaze at a specific point. Arms crossed against the chest was an instruction given by Secomb et al. (2015) citing the protocol of Hasson et al. (2004), while McGuigan et al. (2006) asked participants to hold a light weight (1.0 kg) over their shoulders.

#### Arm and Feet Information

Most of the CMJ and SJ tests were performed either with both hands positioned on the hips (number of articles = 41) or the waist (number of articles = 2). The arms placed in an akimbo position was also used in some works (number of articles = 4). Eight articles did not describe the hands/arms position and only stated that swinging of the arms was not permitted. However, an arm swinging movement was permitted in some CMJ-related research (number of articles = 16). A summary of information regarding the position of the upper limb for the CMJ and SJ is provided in Table 4.

**TABLE 4:** Information regarding the position of the arms and the number of jumps used for analysis.

**Information took in examination**	**Authors**	**Number of studies**
**Arm position**		
Hands positioned on the hips	Alvarez-San Emeterio and Gonzalez-Badillo, 2010; Buchheit et al., 2010; Moliner-Urdiales et al., 2010; Alvarez-San Emeterio et al., 2011; Cortis et al., 2011; Quagliarella et al., 2011; Carvalho et al., 2012; Klusemann et al., 2012; Aloui et al., 2013; Hoshikawa et al., 2013; Hydren et al., 2013; Çakir-Atabek, 2014; Comfort et al., 2014; Lago-Penas et al., 2014; Paradisis et al., 2014; Sekulic et al., 2014; Franco-Márquez et al., 2015; Holden et al., 2015; Saez de Villarreal et al., 2015; Till and Jones, 2015; Lesinski et al., 2016; Loturco et al., 2016; Brännström et al., 2017; Cunha et al., 2017; Gonzalo-Skok et al., 2017; Thomas et al., 2017a, b; Turner et al., 2017; Weakley et al., 2017; Bouteraa et al., 2018; Fischetti et al., 2018, 2019; Hammami R. et al., 2018; Maciejewski et al., 2018; Pino-Ortega et al., 2018; Pojskic et al., 2018; Gavanda et al., 2019; Gonzalez-Garcia et al., 2019; Greco et al., 2019; Paul et al., 2019	41
Hands positioned on the waist	Maio Alves et al., 2010; Carvalho et al., 2012	2
Akimbo position	Grgantov et al., 2013; Lesinski et al., 2016; Negra et al., 2016, 2017	4
Swinging of the arms was not permitted	Maio Alves et al., 2010; Dayne et al., 2011; Lehnert et al., 2013; Garcia-Pinillos et al., 2014, 2015; Maly et al., 2015; Makhlouf et al., 2016; Dowse et al., 2017	8
Swing movement was permitted in the CMJ	Kinugasa and Kilding, 2009; Duncan et al., 2013; Grgantov et al., 2013; Hoshikawa et al., 2013; Lehnert et al., 2013; Matthys et al., 2013; Smart and Gill, 2013; Imai et al., 2014; Lyle et al., 2015; Maly et al., 2015; Munivrana et al., 2015; McCormick et al., 2016; Struzik et al., 2017; Gorski et al., 2018; Idrizovic et al., 2018; Uthoff et al., 2018	16
**Number of jumps**		
Two	Cortis et al., 2011; Lehnert et al., 2013; Smart and Gill, 2013; Imai et al., 2014; Lago-Penas et al., 2014; Makhlouf et al., 2016; Weakley et al., 2017	7
Three	Chelly et al., 2009; Kinugasa and Kilding, 2009; Wong et al., 2009, 2010; Buchheit et al., 2010; Maio Alves et al., 2010; Granacher and Gollhofer, 2011; Granacher et al., 2011; Carvalho et al., 2012; Muehlbauer et al., 2012; Aloui et al., 2013; BenOunis et al., 2013; Duncan et al., 2013; Hammami M. A. et al., 2013; Hoshikawa et al., 2013; Hydren et al., 2013; Matthys et al., 2013; Çakir-Atabek, 2014; Comfort et al., 2014; Garcia-Pinillos et al., 2014, 2015; Paradisis et al., 2014; Holden et al., 2015; Lyle et al., 2015; Till and Jones, 2015; Lesinski et al., 2016; McCormick et al., 2016; Brännström et al., 2017; Cunha et al., 2017; Dowse et al., 2017; Gonzalo-Skok et al., 2017; Hammami M. A. et al., 2017; Hammami M. et al., 2017, 2018; Thomas et al., 2017a, b; Bouteraa et al., 2018; Fischetti et al., 2018, 2019; Maciejewski et al., 2018; Pojskic et al., 2018; Gavanda et al., 2019; Gonzalez-Garcia et al., 2019; Greco et al., 2019; Hale et al., 2019; Martin et al., 2019; Paul et al., 2019	47
Above three	Alvarez-San Emeterio and Gonzalez-Badillo, 2010; Alvarez-San Emeterio et al., 2011; Quagliarella et al., 2011; Klusemann et al., 2012; Franco-Márquez et al., 2015; Saez de Villarreal et al., 2015; Loturco et al., 2016; Pino-Ortega et al., 2018	8
**Jump/s to be considered for analysis**		
Average of the jumps	Garcia-Pinillos et al., 2015	1
Out of five trials, lowest and highest were excluded and middle values averaged	Alvarez-San Emeterio and Gonzalez-Badillo, 2010; Alvarez-San Emeterio et al., 2011; Franco-Márquez et al., 2015; Saez de Villarreal et al., 2015	4
Highest jump	Chelly et al., 2009; Kinugasa and Kilding, 2009; Wong et al., 2009, 2010; Maio Alves et al., 2010; Cortis et al., 2011; Granacher and Gollhofer, 2011; Granacher et al., 2011; Klusemann et al., 2012; Muehlbauer et al., 2012; Aloui et al., 2013; BenOunis et al., 2013; Hammami M. A. et al., 2013; Hoshikawa et al., 2013; Hydren et al., 2013; Lehnert et al., 2013; Matthys et al., 2013; Smart and Gill, 2013; Çakir-Atabek, 2014; Garcia-Pinillos et al., 2014; Imai et al., 2014; Lago-Penas et al., 2014; Paradisis et al., 2014; Lyle et al., 2015; Till and Jones, 2015; Makhlouf et al., 2016; McCormick et al., 2016; Cunha et al., 2017; Dowse et al., 2017; Gonzalo-Skok et al., 2017; Hammami M. A. et al., 2017; Hammami M. et al., 2017, 2018; Struzik et al., 2017; Thomas et al., 2017a; Bouteraa et al., 2018; Fischetti et al., 2018, 2019; Maciejewski et al., 2018; Pojskic et al., 2018; Gavanda et al., 2019; Gonzalez-Garcia et al., 2019; Greco et al., 2019; Hale et al., 2019; Martin et al., 2019; Paul et al., 2019	46

#### Number of Jumps Performance Trials and Result Analysis

The number of trials proposed (Table 4) and the results taken for statistical analysis were either two (number of articles = 7), three (number of articles = 47), or more than three (number of articles = 8). Some studies (number of articles = 1) used the mathematical average of the individual jump trials. Alternatively, out of five trials, the lowest and highest values were excluded averaging only the middle measurements (number of articles = 4). Most studies (number of articles = 46) only considered the highest jump.

#### Devices Used

Several measurement devices were employed for the jump assessment, both to measure and estimate the jump performance. Between the devices that measure the VJ performance based on the center of mass, there are the force plates (number of articles = 25). Between the devices that estimate the performance through the flight time there are: the motion caption system (number of articles = 5), photoelectric cell systems (number of articles = 36), contact mats (number of articles = 19), vanes (number of articles = 10), jump mats (number of articles = 7), contact platforms (number of articles = 7), accelerometer-based systems (number of articles = 6), and linear position transducers (number of articles = 1). More details are given in Tables 1–3.

### Jump Phases Identification

The present review based the jump description and analysis according to the jump phases definition proposed by Bobbert et al. (1996): the starting position; the start of push-off; the toe-off; and the apex of the jump. Additionally, we identified the landing phase.

For the CMJ, the majority of studies (number of articles = 30) agreed in defining the starting position as a standing posture. Some authors provided more details about the position of the lower limbs, suggesting that participants maintain straight legs (Lago-Penas et al., 2014) or position their feet shoulder-width apart (Holden et al., 2015).

The SJ starting position was described by 17 authors as a squat position with knees flexed at 90° and by four authors as a semi-squatting position with knees bent at 90° using a ruler as measurement (Maciejewski et al., 2018). Some researchers required participants to remain in the squat position for either 3 (Comfort et al., 2014; Dowse et al., 2017; Pino-Ortega et al., 2018) or 2 s (Maciejewski et al., 2018) before executing the second phase, on command, i.e., the jump phase.

The CMJ push-off is described as a downward movement without an indication of the depth (number of articles = 26). Some authors indicated that the knee angle had to reach 90° (number of articles = 18) before starting the jump. A limited number of authors provided information regarding the speed of the downward movement, i.e., that it had to perform with a rapid descend (Alvarez-San Emeterio and Gonzalez-Badillo, 2010; Alvarez-San Emeterio et al., 2011; Negra et al., 2016, 2017; Dowse et al., 2017). Regarding the push-off phase for the SJ, most studies performed the jump without a CM (number of articles = 14).

The toe-off phase was described as a maximal effort, i.e., as high as possible (29 works related to the CMJ and 11 works related to the SJ).

For the apex of the jump phase, a requirement of both the CMJ and the SJ was that the participants maintain extended legs (number of articles = 11).

Likewise, to provide reliable results during the execution of the jump landing, standardization is required (Borras et al., 2011). Descriptions of the landing phase were similar for the CMJ and the SJ, with most works reporting a fully extended knee landing (number of articles = 8). Similarly, six works required participants to land without legs flexed. Landing with the toes on the same spot as the take-off (number of articles = 4) and in an upright position (number of articles = 1) were other variants of the instructions given to the participants.

To emphasize the use of the leg extensors, participants were asked to maintain the torso in an upright position (Moliner-Urdiales et al., 2010; Cortis et al., 2011).

A summary of information regarding each CMJ and SJ phase is provided in Table 5.

**TABLE 5:** Information regarding the jump phases.

**Phase of the jump**	**Authors**	**Number of studies**
**Starting position CMJ**		
Standing position	Chelly et al., 2009; Girard and Millet, 2009; Wong et al., 2009, 2010; Alvarez-San Emeterio and Gonzalez-Badillo, 2010; Maio Alves et al., 2010; Moliner-Urdiales et al., 2010; Alvarez-San Emeterio et al., 2011; Cortis et al., 2011; Granacher and Gollhofer, 2011; Granacher et al., 2011; Muehlbauer et al., 2012; BenOunis et al., 2013; Garcia-Pinillos et al., 2014; Lago-Penas et al., 2014; Paradisis et al., 2014; Sekulic et al., 2014; Holden et al., 2015; Loturco et al., 2016; Negra et al., 2016, 2017; Hammami M. et al., 2017; Weakley et al., 2017; Bouteraa et al., 2018; Gorski et al., 2018; Hammami M. et al., 2018; Hammami R. et al., 2018; Idrizovic et al., 2018; Pino-Ortega et al., 2018; Pojskic et al., 2018	30
**Starting position SJ**		
Squat position with a knee flexion of 90°	Chelly et al., 2009; Maio Alves et al., 2010; Dayne et al., 2011; Carvalho et al., 2012; BenOunis et al., 2013; Grgantov et al., 2013; Çakir-Atabek, 2014; Lesinski et al., 2016; Loturco et al., 2016; Negra et al., 2016; Hammami M. et al., 2017, 2018; Bouteraa et al., 2018; Fischetti et al., 2018, 2019; Hammami R. et al., 2018; Pojskic et al., 2018	17
Semi-squat position (knees bent at 90°)	Girard and Millet, 2009; Moliner-Urdiales et al., 2010; Maciejewski et al., 2018; Greco et al., 2019	4
**Push-off CMJ**		
Downward movement without indication on the depth	Girard and Millet, 2009; Alvarez-San Emeterio and Gonzalez-Badillo, 2010; Buchheit et al., 2010; Moliner-Urdiales et al., 2010; Wong et al., 2010; Cortis et al., 2011; Granacher and Gollhofer, 2011; Granacher et al., 2011; Carvalho et al., 2012; Muehlbauer et al., 2012; Hoshikawa et al., 2013; Smart and Gill, 2013; Çakir-Atabek, 2014; Holden et al., 2015; Saez de Villarreal et al., 2015; Till and Jones, 2015; Lesinski et al., 2016; Loturco et al., 2016; McCormick et al., 2016; Cunha et al., 2017; Gonzalo-Skok et al., 2017; Sawczuk et al., 2017; Thomas et al., 2017a, b; Weakley et al., 2017; Pojskic et al., 2018	26
Knee flexion angle to reach 90°	Chelly et al., 2009; BenOunis et al., 2013; Grgantov et al., 2013; Hammami M. A. et al., 2013; Comfort et al., 2014; Lago-Penas et al., 2014; Paradisis et al., 2014; Sekulic et al., 2014; Garcia-Pinillos et al., 2015; Brännström et al., 2017; Hammami M. A. et al., 2017; Hammami M. et al., 2017, 2018; Bouteraa et al., 2018; Hammami R. et al., 2018; Idrizovic et al., 2018; Pino-Ortega et al., 2018; Greco et al., 2019	18
**Push-off SJ**		
Squat jump position	Moliner-Urdiales et al., 2010; Hammami M. A. et al., 2013, 2017; Hoshikawa et al., 2013; Comfort et al., 2014; Lesinski et al., 2016; Thomas et al., 2017a; Bouteraa et al., 2018; Maciejewski et al., 2018; Pino-Ortega et al., 2018; Pojskic et al., 2018	11
**Toe-off CMJ**		
Maximal effort, i.e., as high as possible	Alvarez-San Emeterio and Gonzalez-Badillo, 2010; Buchheit et al., 2010; Faigenbaum et al., 2010; Maio Alves et al., 2010; Alvarez-San Emeterio et al., 2011; Cortis et al., 2011; Granacher and Gollhofer, 2011; Granacher et al., 2011; Carvalho et al., 2012; Klusemann et al., 2012; Muehlbauer et al., 2012; Aloui et al., 2013; Duncan et al., 2013; Hoshikawa et al., 2013; Smart and Gill, 2013; Comfort et al., 2014; Garcia-Pinillos et al., 2014; Imai et al., 2014; Sekulic et al., 2014; Holden et al., 2015; Saez de Villarreal et al., 2015; Dowse et al., 2017; Thomas et al., 2017a, b; Weakley et al., 2017; Hammami R. et al., 2018; Pino-Ortega et al., 2018; Greco et al., 2019; Martin et al., 2019	29
**Toe-off SJ**		
A maximal effort, i.e., as high as possible	Maio Alves et al., 2010; Dayne et al., 2011; Carvalho et al., 2012; Grgantov et al., 2013; Negra et al., 2016; Dowse et al., 2017; Thomas et al., 2017a; Fischetti et al., 2018, 2019; Gorski et al., 2018; Greco et al., 2019	11
Not to perform a CM	Chelly et al., 2009; Girard and Millet, 2009; BenOunis et al., 2013; Grgantov et al., 2013; Çakir-Atabek, 2014; Loturco et al., 2016; Hammami M. et al., 2017, 2018; Fischetti et al., 2018, 2019; Maciejewski et al., 2018; Pino-Ortega et al., 2018; Pojskic et al., 2018; Greco et al., 2019	14
Fast extension of the legs	Negra et al., 2016, 2017; Pino-Ortega et al., 2018; Pojskic et al., 2018	4
**Apex of the jump**		
Maintenance of extended legs CMJ	Chelly et al., 2009; Klusemann et al., 2012; Hammami M. et al., 2017, 2018; Sawczuk et al., 2017; Struzik et al., 2017; Turner et al., 2017; Gavanda et al., 2019; Gonzalez-Garcia et al., 2019	9
SJ	Chelly et al., 2009; Greco et al., 2019	2
**Landing**		
Fully extended knee landing	Buchheit et al., 2010; Cortis et al., 2011; Grgantov et al., 2013; Hammami M. et al., 2017, 2018; Turner et al., 2017; Maciejewski et al., 2018; Pino-Ortega et al., 2018	8
Without any leg flexion	Imai et al., 2014; Gonzalo-Skok et al., 2017; Fischetti et al., 2018, 2019; Gavanda et al., 2019; Greco et al., 2019	6
With toes on the same spot as the take-off	Fischetti et al., 2018, 2019; Pojskic et al., 2018; Greco et al., 2019	4
Upright position	Saez de Villarreal et al., 2015	1

## Discussion

The main finding of this review is that results and recommendations for both the CMJ and the SJ published in the literature are derived using a vast variety of testing methods and devices to evaluate lower body muscular strength in adolescents. It is questionable, therefore, whether results and, where applicable, normative jump height values, recommended for adolescents and used to assess PF are comparable. Therefore, it is necessary to create SOPs for CMJ and SJ tests that can be used in the context of health promotion and health investigations.

With this in mind, we recommend participants start the CMJ from an erect standing position with a straight torso, knees fully extended, with hands-on-hips and feet shoulder-width apart. We also recommend maintaining this position for at least 2 s before the descending phase. The CMJ push-off phase should be characterized by a downward movement until the knee angle reaches 90° and this should be visually inspected by the examiner and where possible, the use of accelerometer that emits audio feedback when the angle is reached (Cular et al., 2018). Instructions for the toe-off phase should explicitly state that it has to be performed with a maximal effort. Furthermore, during the apex of the jump, participants have to keep their legs fully extended. Finally, the landing phase has to occur with both feet together and with knees fully extended.

The SJ starting position is recommended with a knee flexion angle of 90°, torso straight, hands-on-hips, and feet shoulder-width apart. This position should be maintained for 2 s before jumping. The push-off phase has to be executed avoiding any kind of counter-movement. As with the CMJ test, instructions for the toe-off phase should explicitly state that it has to be executed with maximal effort. During the apex of the jump phase, participants should keep their legs fully extended. The landing phase has to occur with both feet together in an upright position with knees fully extended.

Finally, even though different kinds of shoe material can result in artificially deflated jump power and height measurement (LaPorta et al., 2013), to avoid injuries for both CMJ and SJ tests, these should not be performed barefoot (in case of test/retest participants are required to wear the same shoes).

Regarding the measurement device, a photoelectric system is the most commonly employed technology, is less costly, and is very user-friendly. We, therefore, recommend it a part of the SOPs. Furthermore, the equation *H* = *g*^∗^*t*^2^/8 [*H*: VJ height (m); *t*: flight time (s); *g* is 9.81 m/s^2^] presents high coefficients of determination in the prediction of the VJ height (Attia et al., 2017), and is consequently suggested. Five jumps should be performed during the testing session for both CMJ and SJ, with a 1-min passive rest between jumps to ensure muscular recovery. Due to possible learning effects and consequently higher jump performances, only the best jump should be used for further analysis. Furthermore, we recommend starting the CMJ, and SJ testing session with a standardized warm-up as this can influence jump performance (i.e., a short warm-up can improve the jump height, while a high-intensity plyometric protocol deteriorates the performance) (Romero-Franco and Jimenez-Reyes, 2017). Stretching also seems to potentially cause injury rather than prevent it (Shrier, 1999). Our advice is to perform the same standardized warm-up protocol before any VJ. An example of a suitable warm-up protocol has been suggested by Pinfold et al. (2018). This comprises of two sets of the following exercise: (a) standing on one leg and nod head gently for 30 s; (b) single leg airplane squat with hip thrust (20 repetitions); (c) single leg airplane squat with trunk rotation (20 repetitions); (d) single leg airplane squat with a black theraband resistance applied to the knee that includes trunk rotation with a dumbbell held in hand (10 repetitions); (e) monster walk with a black theraband resistance positioned around the forefoot, forward, and backward (3 m each way); (f) monster walk with a black theraband positioned around the forefoot, side-to-side, i.e., left and right (3 m each way). A summary of the first part of the section “Discussion” can be seen in Table 6.

**TABLE 6:** Standard operating procedures proposed for the countermovement jump (CMJ) and the squat jump (SJ).

**Phase**	**CMJ**	**SJ**	
Starting position	Erect position with trunk straight. Knee angle of 180°. Feet shoulder width apart. Maintain the position for at least 2 s	Squat position with trunk straight. Knee angle flex at 90°. Feet shoulder width apart. Maintain the position for at least 2 s	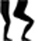
Push-off	Downward movement until the knees angle are flexed (approximately) 90°	No CM	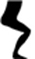
Toe-off	Maximal effort and explosive VJ	Jump vertically as high as possible	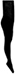
Apex of the jump	Maintain legs extended	Maintain legs extended	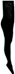
Landing	Feet together. Knees extended at an angle of about 180°	Feet together. Knees extended at an angle of about 180°	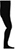
Warm-up suggested	Two sets of the following exercise: (a) standing on one leg and nod head gently for 30 s; (b) single leg airplane squat with hip thrust (20 repetitions); (c) single leg airplane squat with trunk rotation (20 repetitions); (d) single leg airplane squat with a black theraband resistance applied to the knee that includes trunk rotation with a dumbbell held in the hand (10 repetitions); (e) monster walk with a black theraband resistance positioned around the forefoot, forward, and backward (3 m each way); (f) monster walk with a black theraband positioned around the forefoot, side-to-side, i.e., left and right (3 m each way) (Pinfold et al., 2018)		
Hands position	On hips		
Barefoot	No		
Number of jumps	Best of 5		
Rest time	1 min between		
Measurement device	Photoelectric system		
Jump suggested	CMJ		

Concerning the proposed SOPs testing method, the upright position of the torso during the starting position phase for both jumps emphasizes the use of the leg extensors (Moliner-Urdiales et al., 2010; Tounsi et al., 2015). Importantly, this upright position prevents the inclination of the torso segment, a common mistake during the jump performance. In the case of a reduction of forwarding torso inclination by 50%, this can result in a 13% increase of the maximal power (Vanrenterghem et al., 2008). In contrast, hip extensors, upper body, and thigh muscles reduce their contribution on the jump performance when the torso is in a vertical position during the push-off phase, and the plantar flexors contribute mainly to the positive work while knee and hip joint muscles cannot contribute to this positive work (Kopper et al., 2012). In a simulation model, Blache and Monteil (2014) demonstrated that a non-consideration of the erector spinae muscle contribution resulted in a ∼15% reduced SJ height and, if a torso inclination was restricted, the anticipated movement and higher knee joint torque development was possible which resulted in a higher maximal power (Vanrenterghem et al., 2008). A standardized position of the knee angle in the SOPs for the CMJ and the SJ is required during the push-off phase, due to the impact it can have on either increasing or decreasing jump height (Krahenbuhl and Pangrazi, 1983; Gheller et al., 2015) caused by the hip and ankle working differently (Hara et al., 2006, 2008). The execution of the VJ with a lower knee angle compromises the jump performance as a deeper squat starting position results in a higher jump, maximum force, and power output (Gheller et al., 2015). For this precise reason, the SJ has to be carefully monitored. In addition, participants tend to perform a small-amplitude CM (Bobbert et al., 1996; Hasson et al., 2004) and jumps with a CM should be discarded. As the SJ is a purely explosive VJ, some researchers (Hasson et al., 2004; Fitzgerald et al., 2018) argued that the SJ could not be influenced, and consequently performing the SJ with a standardized knee angle or a self-selected jump, apparently present no meaningful difference (Fitzgerald et al., 2018). Based on the discussion above, we strongly advocate the need for the standardized starting position of a 90^*o*^ knee flexion, feet shoulder-width apart, hands-on-hips, and with a straight torso.

Furthermore, this standardized arm position avoids the contribution of the upper limbs as well as coordinative issues as a confounding variable which, as a result of the shoulder, elbow, hip, and ankle muscles working together, can impact on jump performances between 8 and 11% (Harman et al., 1990; Lees et al., 2004; Hara et al., 2006, 2008). Also, the instruction to maintain both legs fully extended starting from the toe-off to the landing phase (i.e., for the entire duration of the apex of the jump phase) is of crucial importance as this can affect the accuracy of the flight time (Borras et al., 2011). Likewise, the landing phase also has to be standardized to obtain equal results during the execution of the jumps (Borras et al., 2011). According to Bui et al. (2015), there are different factors such as the landing with the feet nearly flat or with the legs bent that can alter the flight time, altering the calculated jump height.

Therefore, it is important to land with straight legs, on the forefoot (Bui et al., 2015) and at the same time to amortize the movement because a stiffer technique increases the risk of injuries (Aerts et al., 2013).

Regarding the devices used, a video analysis technique that measures the displacement of the center of body mass from the standing position to the highest vertical point has been proposed as a gold standard (Aragon-Vargas, 2000). However, this specialized equipment is costly, difficult to calibrate, and transport but also requires a complex procedure to obtain data (Aragon-Vargas, 2000). Less expensive, easy to use devices are contact mats which detect the flight time. These have been reported to produce highly reliable and valid results (Markovic et al., 2004). Force plates derive jump height from the flight time, and they can measure the velocity at take-off (Mcgown et al., 1990; Kibele, 1998; Lara et al., 2006). Furthermore, force plates accurately assess ground reaction forces, and can thus provide a preferred solution in pediatric populations (Fricke et al., 2006). Compared to force plates, photoelectric cell systems present a similar level of validity and they provide excellent test–retest reliability for the estimation of the jump height (Glatthorn et al., 2011). Vertec devices (Sports Imports, Hilliard, OH, United States) are also valid (Leard et al., 2007) and reliable (Young et al., 1997) even though not recommended to use for different reasons. Firstly, the measurement device consists of a metal stand and a height scale composed of color-coded vanes that are displaced by the participant when jumping (Klarova, 2000) which requires the use of the arms. Secondly, and in contrast to force plates, the Vertec device (Sports Imports, Hilliard, OH, United States) does not demonstrate an accurate representation of jump height (Buckthorpe et al., 2012).

Claudino et al. (2017) analyzed the average of five CMJs, but, as opposed to the researchers that use jumps to investigate fatigue or super-compensation effects, we recommend to perform the same number of jumps and to use only the best performance.

The proposal of creating SOPs is supported in the literature by researchers who attempted to develop normative gender or country-related VJ test data (Taylor et al., 2010; Holden et al., 2015; Ramirez-Velez et al., 2017). However, considering the different factors that influence maximal jump height performance (e.g., different test methods or assessment criteria), it is unclear whether results are generally comparable with other populations. Furthermore, Claudino et al. (2017) proposed the CMJ to monitor the neuromuscular status using the average of five jump heights but different jumping methods thus compromising the possibility of comparing the VJ. The above further highlights the need for the development of SOPs, which offers researchers a more rigorous and robust test approach. Indeed, it has to be considered that the developed CMJ test protocol by Bosco et al. (1983) was used in only 5 out of the reviewed 102 original works (Table 4). A similar situation applies to the SJ test whereby the two developed protocols by Bosco et al. (1983) and Bosco (1994) were fully replicated in only 3 of the considered 46 works (Table 5).

### Strengths and Limitations

The strength of this review is the stipulation of SOPs for both the CMJ and the SJ test to facilitate the evaluation of the lower limb muscular strength, in a public health context, for adolescents. If these are followed, future communications, sharing of data, result comparisons, and the development of normative data could be made easier and, importantly, these procedures should be more effective in assessing adolescents’ PF. With such a vast variety of testing methods and measurement devices used, results, in fact, are not comparable which present a significant limitation of this review as it was not possible to perform a meta-analysis. The argument above is the rationale for the choice of a quantitative analysis approach of this review. Future works are recommended to review normative data using the stipulated SOPs. A second limitation of the present work is that, due to the mixed samples within the studies analyzed, gender was not considered. Future works should therefore extend their investigation to males, females, and other age groups.

## Conclusion

The present review considered the variety of CMJ and SJ testing method procedures published in the literature, making it impossible to identify standard procedures. Consequently, SOPs for both CMJ and SJ tests have been provided in Table 6 and these are strongly recommended to researchers and health practitioners alike. It is, however, always preferred to study the context first before proposing one protocol over another, especially in the context of sporting performance.

## Author Contributions

LP, APal, and AB developed the research concept and study design. LP, BK, and GM performed the literature review, and data analysis and interpretation. LP and APal performed the data collection. LP, BK, GM, APao, and GD wrote the manuscript. All authors contributed to the revision and approved the submitted version of the manuscript.

## Conflict of Interest

The authors declare that the research was conducted in the absence of any commercial or financial relationships that could be construed as a potential conflict of interest.
